# Children with medical complexity and paediatric palliative care: a retrospective cross-sectional survey of prevalence and needs

**DOI:** 10.1186/s13052-021-01059-8

**Published:** 2021-05-12

**Authors:** S. Amarri, A. Ottaviani, A. Campagna, L. De Panfilis, on behalf of Emilia Romagna paediatric palliative care working group, I. Cortina, E. Sani, P. Bertolini, E. Bianchi, V. Caldarelli, G. Poggi, M. Riva, C. Locatelli, F. Melchionda, M. C. Mondardini, S. Soffritti, E. Mazzoni, L. Serra, L. Gulmini, S. Bertelli, M. Fornaro, A. Magistà, C. Gabriele

**Affiliations:** 1 Fondazione Hospice MT. C. Seràgnoli, Via Marconi 43/45 - 40010, BO Bentivoglio, Italy; 2 Direzione Generale Cura della Persona, Salute e Welfare, Servizio Assistenza Ospedaliera, Bologna, Italy; 3 Bioethics Unit, Azienda USL-IRCCS di Reggio Emilia, Reggio Emilia, Italy

**Keywords:** Children with medical complexity, Hospices, Needs assessment, Palliative care, Paediatrics, Prevalence

## Abstract

**Background:**

Children with medical complexity (CMC) have been defined (Cohen et al., Pediatrics 127: 529–538, 2011.) as an emerging population potentially eligible for PPC. The current study investigated the prevalence of children with medical complexities eligible for a local palliative care network, including a paediatric hospice.

**Methods:**

A retrospective cross-sectional survey has been conducted using children clinical charts from 14 local health authorities of our region (Emilia Romagna, Italy).

**Results:**

The total number of children with life-limiting conditions was 601, with a mean age of 7.4 ± 4.8 years, a prevalence of 8.4/10.000 residents < 19 years of age and a heterogeneous presentation among the provinces in the region. Neurological diseases affect 51% of patients, followed by congenital diseases (21%) and pathologies originating in the perinatal period (6%), while only 4% of the patients had a cancer diagnosis. Patients are dependent from many devices and supports: 32% had a gastrostomy, 22% a respiratory support and 15% of patients had both of them.

**Conclusions:**

Observed regional prevalence of complex needs is lower than that published from other European countries. More research is needed to raise awareness of palliative care for children with medical complexities in order to address specific needs.

## Background

Paediatric Palliative Care (PPC) services have been acknowledged as fundamental for improving the quality of life of children with life-limiting and life-threatening conditions and their families. They have evolved in the past years towards a paediatric subspecialty, encompassing inpatient and outpatient care in hospital along with community-based services [1]. PPC services are increasing worldwide, although models of care may be different and analysis of need is still at an early stage. Whilst some countries, such as the UK, USA, Canada and Australia, have established PPC services, many other countries are still looking for a suitable approach to care children and families with PPC needs in-line with the WHO definition of PPC [2].

The total number of children in need of PPC worldwide each year may be as high as 21 million, with 8 million having problems that require specialist PPC [3].

Children with medical complexity (CMC) have been defined [4] as an emerging population potentially eligible for PPC. They have multiple chronic health problems that affect multiple organs resulting in: functional limitations, high health care utilisation, and the need for medical technology. They are also at high risk of adverse medical, developmental, psychosocial, and family outcomes.

According to the Italian law on Palliative Care (PC) [5], PPC must be provided nationwide in Italy, strengthening the development of regional networks set up by the existing health services and by dedicated structures for PPC services, such as children hospices. Many of the 21 regions have designed a regional network of PPC, and seven dedicated hospices have been opened so far.

Since 2012, the Emilia Romagna Region (RER) (North of Italy) has developed a regional PPC network thanks to a public-private collaboration with a non-profit organization working in the palliative care field since 2001. Currently, a regional PPC day care service and a regional paediatric hospice are under construction.

This paper is a retrospective cross-sectional survey to obtain a snapshot of PPC patients being seen by the existing regional network, to plan the RER PPC health policy, and review the design of the PPC network. It aims to identify the prevalence of CMC at a regional level compared to international data available from the literature. The study is preliminary to the implementation of a regional paediatric hospice.

## Methods

A retrospective cross-sectional survey was done [6], providing the information needed to gather targeted results from which to draw conclusions and make important decisions [7].

The study was designed in collaboration with health care professionals involved in the care of potentially eligible PPC patients in 14 local health authorities within the RER.

In the first step, the research team presented the project to all 14 local health authorities during structured meetings with local health professionals from the paediatric services and units potentially involved with CMC care. After obtained the consent to participate, data have been collected.

Inclusion criteria were:

- Life-limiting or life-threatening conditions included in the the Association for Children’s Palliative Care/Royal College of Paediatrics and Child Health [8] classification:
  - Life-threatening conditions for which curative treatment may be feasible but can fail. PC may be necessary during phases of prognostic uncertainty and when treatment fails
  - Conditions where premature death is inevitable. There may be long phases of intensive treatment aimed at prolonging life and allowing participation in normal childhood activities
  - Progressive conditions without curative treatment options, in which treatment is exclusively palliative and may commonly extend over many years
  - Irreversible but non-progressive conditions causing severe disability leading to susceptibility to health complications and likelihood of premature death. There may be unpredictable and periodic episodes of care
- The child received care either at home or in hospital during 2017
- The child and its family live in the RER
- The child was under the age of 19.

Quantitative data collected through the survey included:

- Personal information e.g. age, sex, city of residence, family composition
- Pathologies with corresponding ICD9 codes (up to 3 concurrent conditions);
- Health service use e.g. date of first contact, date of death, admissions in 2017, which community and medical services they accessed;
- Medical supports e.g. mobilisation aids, ventilator, oxygen, and medical devices e.g. tracheostomy, gastrostomy, urinary catheter
- Evaluation of clinical complexity utilising a recently validated scale: the Assessment of Complex Clinical Assistance Needs in Paediatrics (ACCAPED) Tool [9]. The ACCAPED questionnaire contains information on a range of clinical issues (breathing; nutrition; epilepsy and state of consciousness; skin and tissue integrity; mobility; ability to communicate; sleeping characteristics; faecal continence; medications; pain; and unexpected or unpredictable events that could lead to death) to detect the complexity of clinical needs and the allocation to the appropriate PPC care level. Scores ≤ 29 come from patients typically handled at the primary care level, intermediate scores (30–49) are describing patients that can be referred for a PPC consultation at a specialistic level, while ≥ 50 is indication for full management by a third level PPC centre.
- The number of deaths during 2017 and 2018.

Information was entered into a Microsoft® Excel® spreadsheet which was sent to local health authorities to be completed as per the survey questions and then returned to the researchers. All data were anonymised, and no identifying data were included.

Data analysis of descriptive data was undertaken using Microsoft® Excel® based tools.

Prevalence was calculated by identifying the number of children with CMC per 10,000 residents < 19 years of age.

According to the Italian legislation, a formal ethical approval for this study was not required. Patient data were derived from children clinical charts within the hospital or at home. All data were inserted anonymously into the Excel® spreadsheets, with no identifying data collected.

## Results

All health authorities involved accepted to participate in the survey, collecting data from their medical records. Data were obtained from children’s clinical records for both those being cared for at home and in the hospital. The survey was carried out in 2018, data collected referred to children followed during 2017.

In 2017 there were 4,461,612 people living in the RER with 711,765 < 19 year [10]. The total number of children reported to have a CMC was 601 with an overall prevalence of 8.44 per 10,000 inhabitants < 19 years of age – ranging from 3.31–11.61 in different provinces (Table 1).

**Table 1:** Total and provincial prevalence of CMC children (n/10,000 resident < 19 years of age)

*Province*	*Patients*	*Neoplasm*	*Prevalence (n/10,000 < 19 y)*
Bologna	170	8	10,81
Modena	100	1	8,41
Ravenna	70	5	11,61
Reggio Emilia	70	2	7,39
Parma	67	3	9,39
Rimini	45	1	8,12
Piacenza	38	2	8,65
Forlì-Cesena	21	3	3,31
Ferrara	20	0	4,31
**Total**	**601**	**25**	**8,44**

The total mean age was 7.4 ± 4.8 years (median age 7 years, range 0–18 years). This was the same for the non-oncological patients (7.2 ± 4.80 years) who also had an identical median age, whilst oncological patients were older with a mean age of 9.1 ± 4.5 (median age 10, range 1–17 years). 56% were female and 44% male, 90% of the main caregivers were the parents, and 42% of the children had non-Italian parents.

**Fig. 1:**
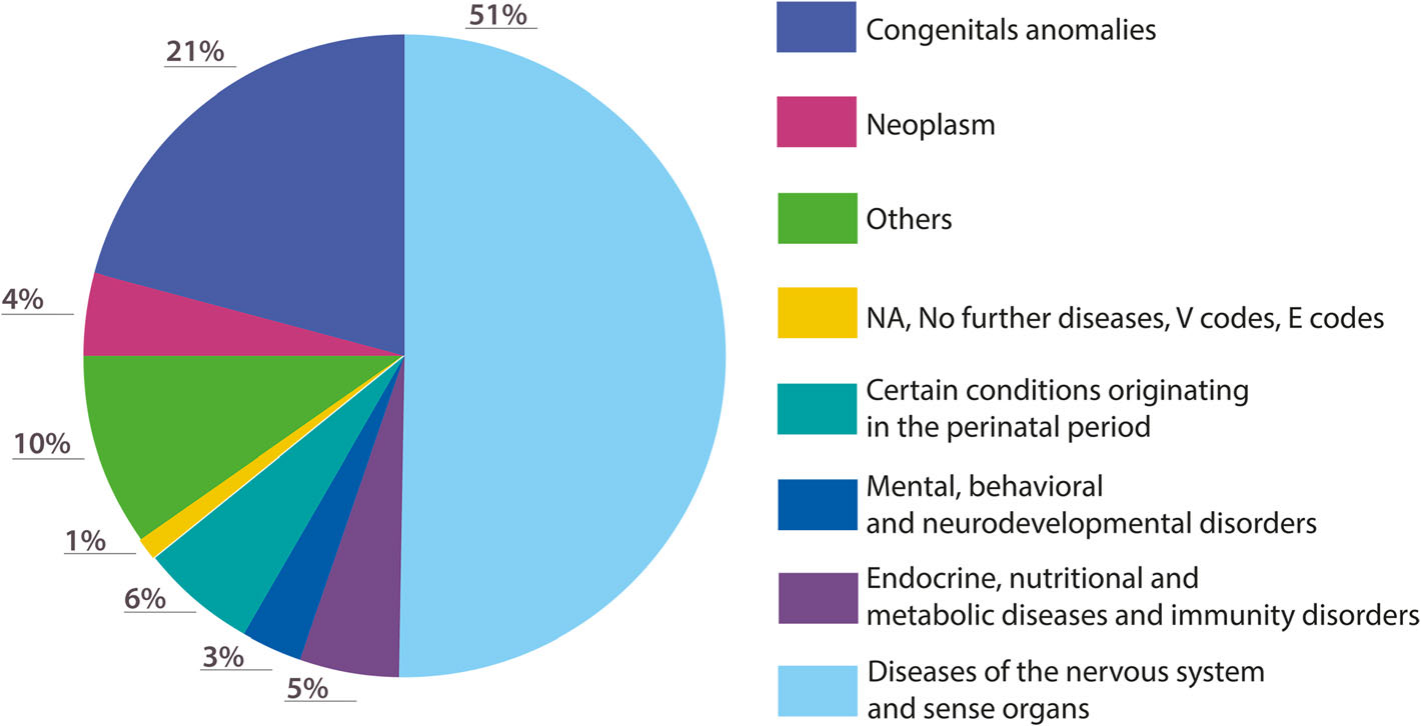
ICD9 main subgroup diagnosis among patients

The total number of conditions reported via the ICD9 codes was 195, which could be divided into eight main subgroups (Fig. 1). Approximately half (51%) of the children had neurological conditions (the single most frequent diagnosis was cerebral palsy (*n* = 116 (19.3%)) and most had neurological symptoms. The second most common group were those with congenital anomalies (21%), 35 (5.8% of total) were chromosomopathies. Only 4.2% (*n* = 25) of children had oncological diseases of whom 9 had tumours of the central nervous system, 3 bone tumours and 9 haematological neoplasms (Table 2).

**Table 2:** Oncological diagnosis (total n. 25)

***Main pathology***	***ICD9 codes***	***Number of cases***
Lymphoma	20,023, 20,268	2
Hodgkin Lymphoma	2019	1
Acute Leukemia	2040, 2041, 2080	5
Lymphangioma	2281	1
Ewing Sarcoma	3342	1
Other Bone Tumors	1708, 1712	2
Connective tissue tumors	1719	2
Neurofibromatosis Type I	23,771	1
Malignant central nervous system tumors	1910,1915, 1916,1917, 1919, 1922	9
Urinary tract tumor	V105	1
Total		25

Twenty-nine deaths occurred during 2017 and 2018, six of whom (mean age 6.17 ± 4.84 years) were oncology patients (24%). The 23 deaths among the non-oncological patients (mean age 6.04 ± 5.58 years) were primarily due to diseases of the central nervous system (70%) and congenital anomalies (17%). A mean of one (±2.2) hospital admission per year was observed in these children with CMC. 70% were urgent admissions with a mean length of hospital stay of 4.5 ± 18.3 days (up to a maximum of 195 days); the mean day hospital attendance was 2.3 ± 11.5 days per year, with a maximum of 201.

**Fig. 2:**
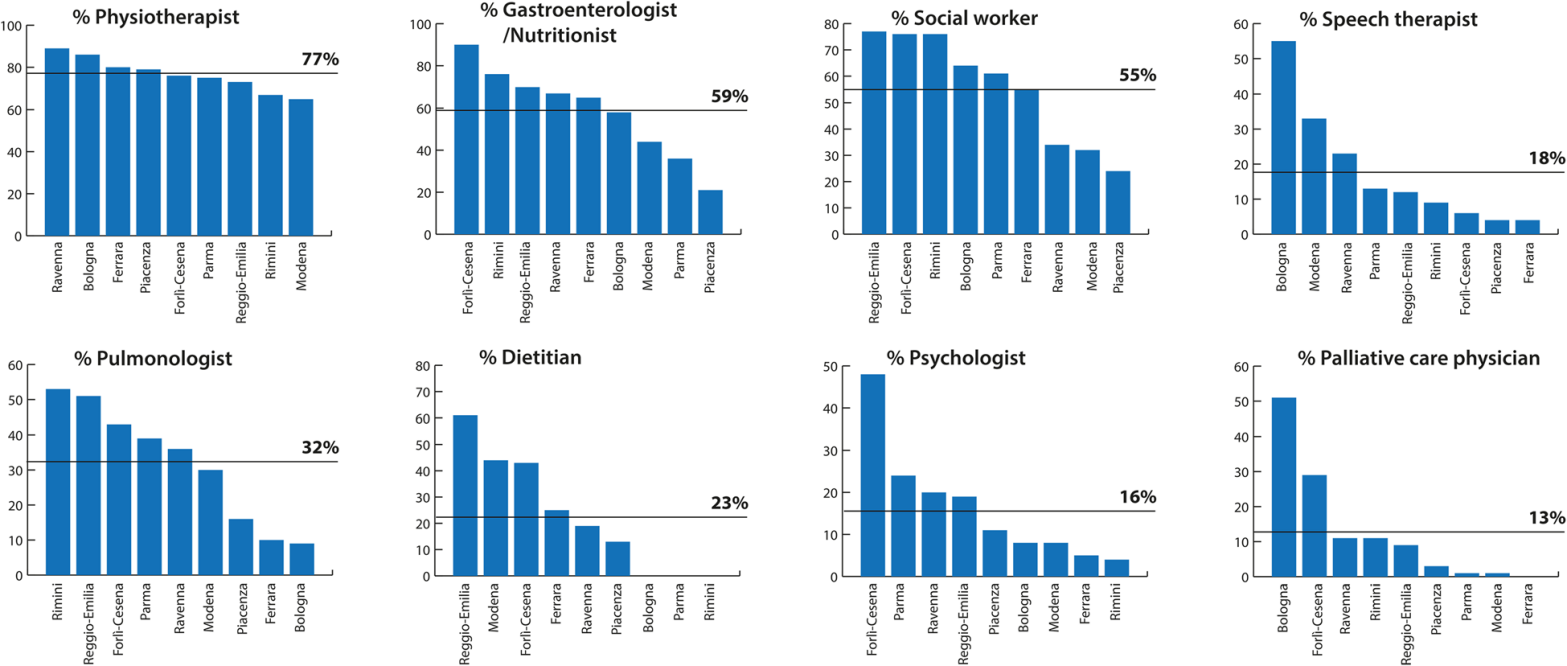
Percentage of children receiving care from different health professionals

The number of health professionals involved in the care of individual children increased as clinical complexity increased and were scattered throughout the region. Figure 2 describes the percentage of children receiving care from different health professionals, with the physiotherapist being the most common, with 76.6% (*n* = 460) of children receiving care from a physiotherapist, while only the minority of children were regularly followed by a PC physician. Regular home care was provided for 45% (*n* = 270) of children and their families.

The majority of children (84% *n* = 505) utilised at least one type of support e.g. mobilisation aids, ventilator, oxygen and 42% (*n* = 252) had at least one medical device. 32% (*n* = 192) of children had a gastrostomy, 11.3% (*n* = 68) were using a mechanical ventilator and 9% (*n* = 54) had a tracheostomy. (Table 3). 15% of children had both nutritional and respiratory supports.

**Table 3:** Medical Aids and Devices among patients

***Aid or Device***	***% patients***
Suction Aspirator	30%
Mobility Aids	71%
Motor Physiotherapy Aids	38,1%
Respiratory Physiotherapy Aids	32,8%
Mechanical Ventilator	11%
Oxygen Therapy/High Flow	19%
Tracheostomy	9%
Monitor/Oximeter	34%
Enteral Nutrition Pump	31%
Gastrostomy	32%
Jejunostomy	2%
Central Venous Catheter/Midline	7%
Urinary catheter	4%

**Fig. 3:**
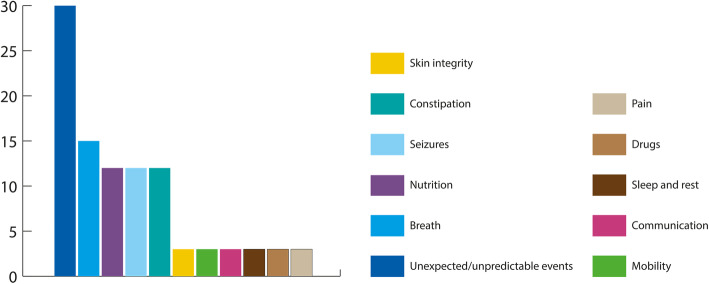
Impact of different ACCAPED domains on patient complexity

Patient clinical complexity was high with a mean ACCAPED score of 51, with 48% of patients showing a score ≥ 50, 30% 30–49 (second category) and 22% ≤29. The different domains of the ACCAPED showed that the presence of potential unexpected/ unpredictable events was the greatest concern (identified in 30% of children, *n* = 180), followed by challenges in breathing, nutrition, seizures and constipation (Fig. 3).

## Discussion

The study investigates the prevalence of CMC in the RER while outlining their PC needs. To our knowledge, this is the first survey in the nation directly involving healthcare professionals to apply definitions and criteria for PPC to their patients. This is also the first survey in a specific region in Italy undertaken prior to opening a new Children’s Hospice.

Our study shows a lower prevalence of CMC, with an overall prevalence of 8.44 per 10,000 inhabitants < 19 years of age. This result is interesting, compared to the need for PPC in other countries with high/upper middle incomes, reported [3, 11] in a range between 20 and 30 children per 10,000 inhabitants < 19 years of age. More recently, a study published in the UK [12] has shown an increase in the number of children to 66.4 per 10,000 children living with life-limiting condition. Our findings are similar to that seen in a nearby region in 2008 when a prevalence of 9.5/10.000 was found [13]. Our different results may depend on two issues. First, when we performed the survey newer and more accurate eligibility criteria [14, 15] were not available. Moreover, there may have been some variation in the understanding of PC from health care professionals involved: as demonstrated by literature the awareness of PPC by health professionals is variable [16].

In addition, our patients have a high average clinical complexity and could represent an estimation of patients potentially referable to a specialist level PCC services, instead of including the complete set of patients eligible to the various level of PPC care. As services become more developed within the region and more professionals are sensitised to PPC, the number identified as having a CMC, and therefore be potentially eligible for PC, may increase.

According to our survey, approximately half (51%) of the children had neurological conditions, the single most frequent diagnosis was cerebral palsy (*n* = 116–19.3%). This result is in line with an extensive study performed in the UK in the years 2011–2012 [13] of children with life-limiting diseases, aiming to identify prevalence of need for palliative and supportive care. It found similar mean age (8 years) and prevalence (8–10 per 10,000 children), with the majority of patients having neurological or congenital diseases. On the contrary, studies from the US [17] and France [18] reported higher oncology prevalence (30 and 26% respectively) whilst in Japan [19] they reported a similar prevalence of oncological diseases (7%). The high prevalence of neurological diseases has already been described in the USA and Canada [20] and highlighted by a recent review on PC for paediatric neurology [21]. A major study in Italy showed more than 300,000 (0–17-year-old) hospital admissions involving 12,000 children eligible for PPC [22]. The study does not give the percentage of oncological patients (who went to hospital mainly for disease management or diagnostic and therapeutic procedures) and non-oncological patients, who were admitted for a variety of diseases or complications (neurological 34%, respiratory 25%, digestive 14%, congenital 4%, perinatal 7%, others 16%).

The high utilisation of medical aids and devices described in our study is in line with other national studies reporting at least two medical aids/devices per child with regards to respiratory, feeding, pain and seizures [23, 24]. Children wih multiple chronic complex condition and neurological impairment, together with the use of technological assistance are known to have higher access to health services and assistance costs [25].

The results presented here have been instrumental in incorporating WHO guidelines for [26] planning our regional service based on actual numbers, and observed needs of local CMC, and will be the basis on which to try to fulfil major PPC challenges over the next year, such as allowing equal access to PPC services, integration between PPC teams and facilitation of continuous care between hospital and community [1]. What is anticipated is a PPC network, covering the entire area of RER and including both home care and hospital care together with one regional children’s hospice, as an efficient and effective model of care. A specialised PPC team will be the reference for network professionals, managing and making the best use of health services in response to specific patients and caregiver needs.

Less severe patients are probably still hidden to our PPC Network, in particular the neonates and premature babies [27].

Perinatal PPC was not included, although it has been recognised internationally and nationally [28] as an important component of PPC, thus having an impact on the prevalence, as the Global Atlas for PC at the end of life identifies 14.64% of children < 15 needing PC at the end-of-life are neonates [29].

According to our results, only 4% of children having cancer. When further examined, these 23 cases were those receiving end-of-life care. Since we mainly measured health services consumption it is likely that we missed many oncological patients with low access to such services, especially as outpatients, although they may have had a potentially poor prognosis and be eligible for early PPC.

Our attempt to describe the complexity was limited: ACCAPED scoring is designed to assess only clinical complexity. We have therefore missed the full picture of patient and his/her family/caregiver complexity: health being only part of a much broader scenario that usually includes school, recreation, community, self-support, advocacy, leadership and financial issues. Such a full approach would provide a proactive, rather than reactive, care so that critical medical and health events are avoided to the greatest extent possible [30].

## Conclusions

This survey provides the basis for an extended application of PPC in RER, starting from an understanding of who, how many and where the children are and who currently cares for them. It has resulted in the setting up of a permanent PPC network that is constantly updating its patient list.

The knowledge of patient characteristics and caregiver needs has been highly valuable in designing and improving the network, because PPC can extend throughout the illness trajectory, and it is therefore important to adopt an integrated model with community-based PPC and family centred care and to properly disseminate PPC throughout the health system.

## Data Availability

The datasets used and analyzed during the current study are available from the corresponding author on reasonable request.

## References

[1] Sisk BA, Feudtner C, Bluebond-Langner M, Sourkes B, Hinds PS, Wolfe J (2020). Response to suffering of the seriously ill child: a history of palliative Care for Children. Pediatrics..

[2] World Health Organization. WHO definition of palliative care for children. Available from URL: https://www.who.int/cancer/palliative/definition/en/. Accessed 12 Oct 2020.

[3] Connor SR, Downing J, Marston J (2017). Estimating the global need for palliative care for children: a cross- sectional analysis. J Pain Symptom Manage.

[4] Cohen E, Kuo DZ, Agrawal R, Berry JG, Bahgat SKM, Simon TD (2011). Children with medical complexity: an emerging population for clinical and research initiatives. Pediatrics..

[5] LEGGE 15 marzo 2010, n. 38. Disposizioni per garantire l'accesso alle cure palliative e alla terapia del dolore. Available from URL: http://www.parlamento.it/parlam/leggi/10038l.htm (2010). Accessed 12 Sept 2020.

[6] Ponto J (2015). Understanding and evaluating survey research. J Adv Pract Oncol.

[7] DeFranzo SE. Main benefit of survey research. https://www.snapsurveys.com/blog/4-main-benefits-survey-research/ (2012). Accessed 18 Sept 2020.

[8] © ACT (Association for Children’s Palliative Care) A Guide to the Development of Children’s Palliative Care Services (Third Edition) England 2009 ISBN 1 898 447 09 8.

[9] Lazzarin P, Giacomelli L, Terrenato I, Benini F. A tool for the evaluation of clinical needs and eligibility to pediatric palliative care: the validation of the ACCAPED scale. Accepted for publication on J Palliat Med. Epub ahead of print 8 July 2020. 10.1089/jpm.2020.0148 32640899

[10] Regione Emilia Romagna, Statistica. Available from: https://statistica.regione.emilia-romagna.it/. Accessed 12 Sept 2020.

[11] Fraser LK, Miller M, Hain R, Norman P, Aldridge J, McKinney PA (2012). Rising national prevalence of life-limiting conditions in children in England. Pediatrics..

[12] Fraser L, Gibson-Smith D, Jarvis S, Norman P, Parslow R. ‘Make every child count’. Estimating current and future prevalence of children and young people with life-limiting conditions in the United Kingdom. Available from URL: https://www.york.ac.uk/media/healthsciences/documents/research/public-health/mhrc/Prevalence%20reportFinal.pdf. Accessed 10 Sept 2020.

[13] Benini F, Ferrante A, Visonà Dalla Pozza L, Trapanotto M, Fachin P (2008). Children’s needs: key figures from the Veneto region, Italy. Eur J Palliat Care.

[14] The Big Study: meeting the needs of life-limited children in the West Midlands. Available from URL: https://www.togetherforshortlives.org.uk/resource/the-big-study/ (2012). Accessed 15 Sept 2020.

[15] Jankovic M, De Zen L, Pelegatta F, Lazzarin P, Bertolotti M, Manfredini L (2019). A consensus conference report on defining the eligibility criteria for pediatric palliative care in Italy. It J Pediatr.

[16] Bogetz JF, Root MC, Purser L, Torkildson C (2019). Comparing health care provider-perceived barriers to pediatric palliative care fifteen years ago and today. J Palliat Med.

[17] Bona K, Bates J, Wolfe J (2011). Massachusetts’ pediatric palliative care network: successful implementation of a novel state-funded pediatric palliative care program. J Palliat Med.

[18] Marty L, Bernard F, Pierre M, Bringuier-Branchereau S, SirventN RA (2017). Recours initial à une équipe régionale ressource de soins palliatifs pédiatriques: étude observationelle prospective. Arch Pediatr.

[19] Ando K, Nabetani M, Yotani N, Sano H (2017). Three years’ experience with first pediatric hospice in Asia. Brain and Development.

[20] Feudtner C, Kang TI, Hexem KR, Friedrichsdorf SJ, Osenga K, Siden H (2011). Pediatric palliative care patients: a prospective multicenter cohort study. Pediatrics..

[21] Lyons-Warren AM (2019). Update on palliative Care for Pediatric Neurology. Am J Hosp Palliat Care.

[22] Benini F, Trapanotto M, Spizzichino M, Lispi L, Visonà L (2010). Ferrante Anna. Hospitalization in children eligible for palliative care. J Palliat Med.

[23] Lazzarin P, Schiavon B, Brugnaro L, Benini F (2018). Parents spend an average of nine hours a day providing palliative care for children at home and need to maintain an average of five life-saving devices. Acta Paediatr Int J Paediatr.

[24] Rusalen F, Agosto C, Brugnano L, Benini F (2018). Impact of the regional pediatric palliative care network on the care of children on long term ventilation: could the availability of a residential solution into the network reduce the duration of intensive care unit staying for these patients?. J Pediatr Intensive Care.

[25] Cohen E, Berry JG, Camacho X, Anderson G, Wodchis W, Guttmann A (2012). Patterns and costs of health care use of children with medical complexity. Pediatrics..

[26] World Health Organization. Integrating palliative care and symptom relief into primary health care: a WHO guide for planners, implementers and managers. World Health Organization. 2018. Available from URL: https://apps.who.int/iris/handle/10665/274559. Accessed 29 Aug 2020.

[27] Younge N, Goldstein RF, Bann CM, Hintz SR, Patel RM, Smith PB (2017). Survival and neurodevelopmental outcomes among Periviable infants. N Engl J Med.

[28] Cavicchiolo ME, Rusalen F, Benini F, Baraldi E, Lago P (2019). Perinatal palliative care: a national survey. Arch Dis Child Fetal Neonatal Ed.

[29] Connor SR, Sepulveda C (eds). Global Atlas of Palliative Care at the End of Life 2014. World Health Organization/Worldwide Hospice Palliative Care Alliance, Geneva/London. Available from URL: http://www.thewhpca.org/resources/global-atlas-on-end-of-life-care. Accessed 20 Aug 2020.

[30] Thienprayoon R, Alessandrini E, Frimpong-Manso M, Grossoehme D (2018). Defining provider-prioritized domains of quality in pediatric home-based hospice and palliative care: a study of the Ohio pediatric palliative care and end-of-life network. J Palliat Med.

